# A case report of possible concurrent vasculitis in vertebral bodies and partial transverse myelitis following COVID-19 vaccination

**DOI:** 10.1097/MD.0000000000030814

**Published:** 2022-09-30

**Authors:** Yanyi Chen, Yuxin Li, Tao Zhan

**Affiliations:** a Department of Integrated TCM and Western Medicine, The First Hospital of Changsha, Changsha, Hunan, China; b Department of Radiology, The First Hospital of Changsha, Changsha, Hunan, China.

**Keywords:** COVID-19 vaccine, demyelinating diseases, transverse myelitis, vasculitis

## Abstract

Cases with organ-specific and systemic vasculitis associated with corona virus disease 2019 (COVID-19) vaccination have been reported. However, acute partial transverse myelitis (APTM) is rare adverse events following received COVID-19 vaccines. To the best of our knowledge, there is no report on vaccine-associated APTM accompanied by possible concurrent vasculitis. Herein we present a case with possible concurrent spinal vasculitis and APTM following the second dose of inactivated COVID-19 vaccine.

**Case summary:**

A 33-year-old man presented with weakness of left lower limb and aberrant sensation of his left lower trunk and limb (from T9 level to toes) for 2 days following receipt of an inactivated COVID-19 vaccine. Remarkable demyelinating lesion at T7 spinal cord was showed by 3.0T magnetic resonance imaging (MRI) scan. Moreover, vertebral bodies of T3-T7 also presented high signal in T-2 weighted imaging (T2WI) accompanied by multiple sites of flowing void effect indicating possible vasculitis. Oligoclonal band was positive in cerebrospinal fluid (CSF) while it was negative in sera. Intravenous methylprednisolone (1 g/d) was administrated for 5 days followed by subsequent dose-tapering prednisone. His limb weakness and aberrant sensation both improved and he was able to walk unaided after treatment. The MRI recheck also showed remarkable improvement on the lesions in spinal cord and vertebral bodies.

**Conclusion:**

this case illustrates the concurrence of possible vasculitis in vertebral bodies and acute transverse myelitis (ATM) following COVID-19 vaccination.

## 1. Introduction

Large-scale immunization is a pivotal strategy for achieving successful control of the pandemic corona virus disease 2019 (COVID-19). As the worldwide vaccination is ongoing, occurrence of various vaccine associated adverse events has been the most extensive concern. Incidences of vaccination-associated vasculitis affecting various organs had been reported.^[1]^ Autoimmune neurological conditions, such as Guillain–Barré syndrome, acute disseminated encephalomyelitis and acute transverse myelitis (ATM), are considered severe post-vaccination adverse events. Acute partial transverse myelitis (APTM) is a rare demyelinating disease with different manifestations and radiological change compared to typical ATM with symmetric symptoms and greater lesion. Although co-occurrence of autoimmune vasculitis and demyelinating diseases including ATM has been noticed,^[2]^ there is no report on APTM accompanied by concurrent vasculitis. Herein we present a case with possible concurrent spinal vasculitis and APTM following the second dose of inactivated COVID-19 vaccine.

## 2. Case presentation

A 33-year-old man presented to our hospital with weakness of left lower limb and aberrant sensation (tightness and numbness) of his left lower trunk and limb (from T9 level to toes) for 2 days. He was transferred to a local hospital on the day of onset. Cervical and thoracic spine magnetic resonance imaging (MRI) (1.5T) scanned in the local hospital showed no remarkable change in the spine. Ten days before admission he received a second dose of inactivated COVID-19 vaccine CoronaVac.

**Figure 1.**
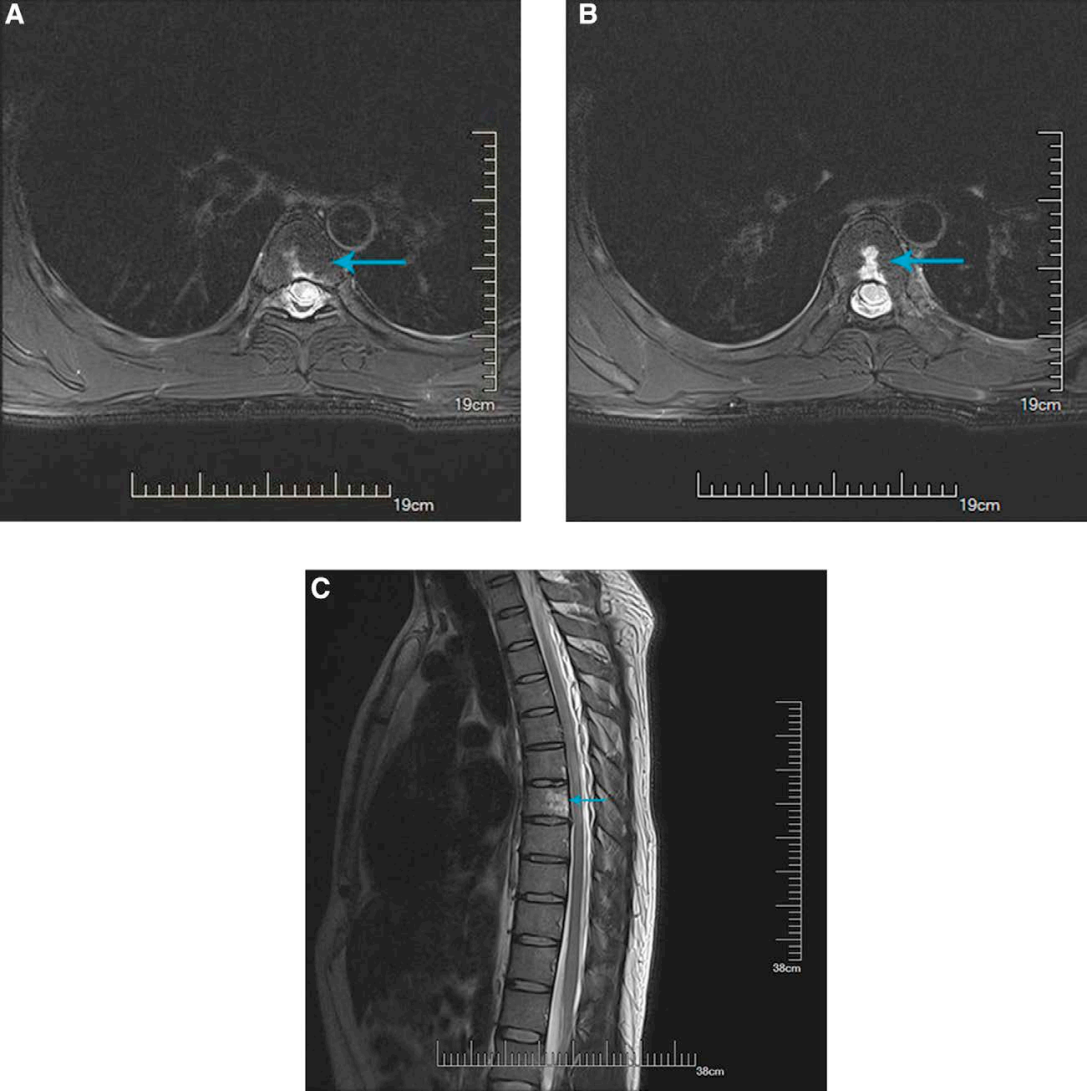
Axial MRI T2WI sequence showing high signal lesion in T7 spinal cord and vertebrates before treatment. MRI = magnetic resonance imaging, T2WI = T-2 weighted imaging.

Initial physical examination revealed unilateral reduced superficial sensation below the T8 level. Slightly spastic monoplegia (grade 4 of muscle strength) and positive Babinski’s sign on the affected limb were objectified. Cranial nerves and cognition were normal. A brain and thoracic spine MRI (3.0T) was performed on the 5th day after onset. We found remarkable aberrant high signal in T-2 weighted imaging (T2WI) by axial scan at T7 spinal cord indicating an acute demyelinating lesion (Fig. 1a–c). Moreover, vertebral bodies of T3-T7 also presented high signal in T2WI accompanied by multiple sites of flowing void effect. No inflammatory change were found in cranial MRI scan. Conventional laboratory tests including blood cell counts, coagulation, liver function, kidney function, electrolytes, plasma glucose, lipids, C-reaction protein and procalcitonin were normal. No remarkable change was identified in routine cerebrospinal fluid (CSF) except for a slightly elevated glucose level (5.42 nmol/L). Serum demyelination-related autoimmune antibodies including anti-AQP4, anti-MOG, and anti-MBP were all negative. CSF immunoglobulin classification test showed a slightly elevated IgA level (5.11 mg/L). Oligoclonal band (OCB) was positive in CSF while it was negative in sera.

**Figure 2.**
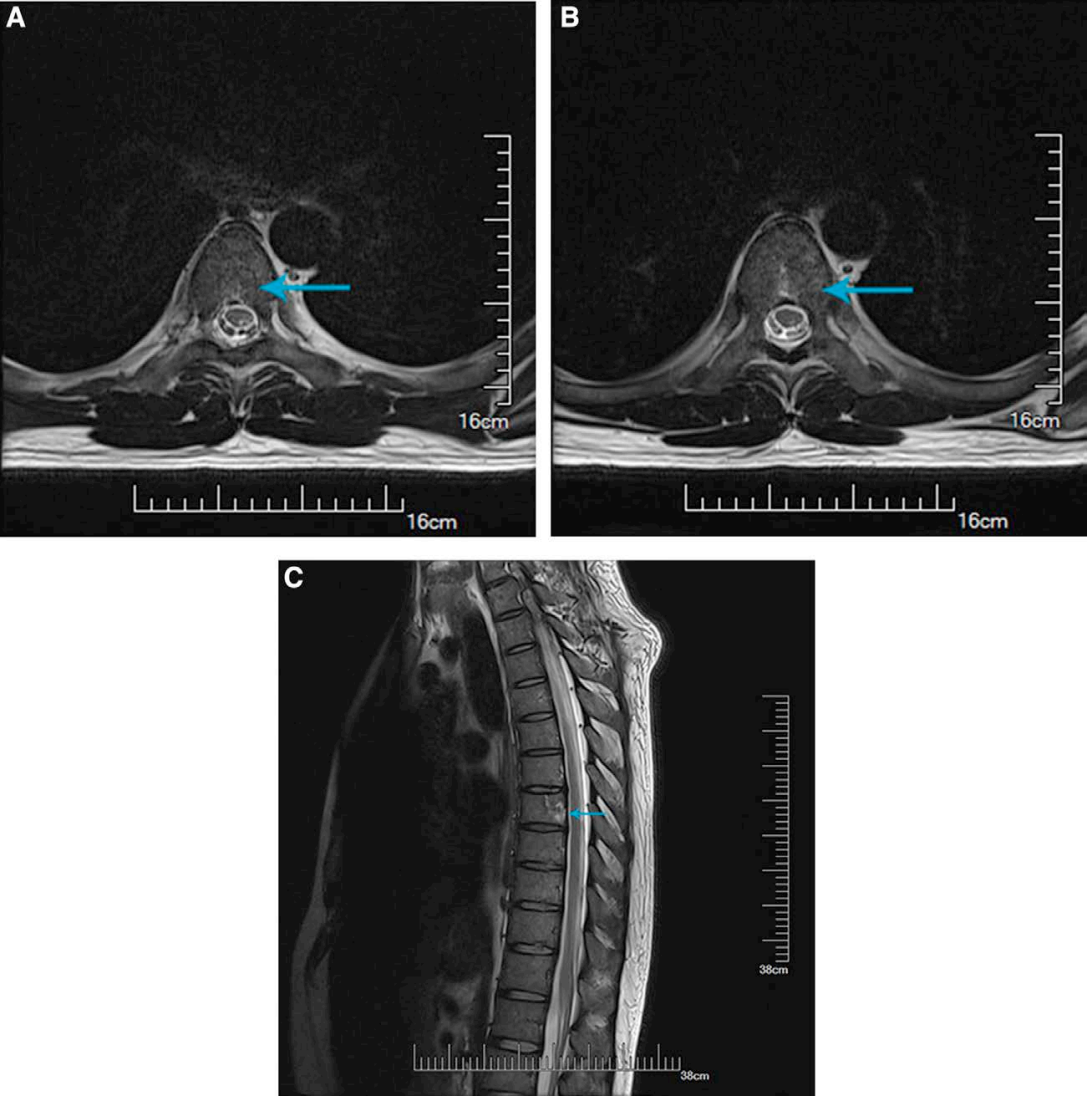
Axial MRI T2WI sequence showing improvement of the high signal lesion in spinal cord and vertebrates after treatment. MRI = magnetic resonance imaging, T2WI = T-2 weighted imaging.

A large dose of intravenous methylprednisolone (1g/d) was administrated for 5 days and subsequent dose-tapering prednisone. In addition, he received movement rehabilitation, acupuncture and Vitamin B1/B12 administration during hospitalization. His symptoms started to improve after 3 days of treatment. On the day discharging, the strength of his left lower limb turned normal and he was able to walk unaided. The aberrant sensation below T8 level was also “obviously relieved.” He discontinued oral steroids on the 30th day after discharge and reported “slight numbness and nearly normal strength of left leg” at telephone follow-up. He had a MRI recheck on the 45th day after discharge. The lesions in spinal cord and vertebral bodies all remarkably improved (Fig. 2a–c).

## 3. Disscussion

Acute non-traumatic lesions simultaneously involving vertebrates and spinal cord are puzzling changes regarding the etiology and diagnosis. In this case, the high signal in T2 and DWI sequence in spinal cord indicate possibility of inflammation or ischemia. However, the MRI characteristics in the vertebrates do not suggest ischemia. The multiple sites of flowing void effect are considered evidence for abnormality of small vessels. CSF analysis does not suggest infection, tuberculosis or malignancies. The concurrence and simultaneous improvement of the lesions following corticosteroids treatment imply a common etiology. Therefore, non-infective inflammation is the most likely etiology in light of medical history, laboratory workups, MRI characteristics and responsiveness to steroids.

Vasculitis has been reported as a complication associated with COVID-19. severe acute respiratory syndrome coronavirus-2 (SARS-CoV-2) infection can induce various vasculitis forms affecting skin, gastrointestinal tract, aorta, coronary arteries, mesentery, lung, kidney, liver and central nerve system.^[3]^ Systematic vasculitis such as Kawasaki disease and Kawasaki-like disease spectrum associated with SARS-CoV-2 infection were also reported in children and young adults.^[4]^ Endothelial inflammation following viral infection is recognized as the initiation of COVID-19 associated vasculitis.^[5]^ Cases with absence of SARS-CoV-2 RNA at where vasculitis occurs indicating that autoimmune reaction may play an important role in the formation of vasculitis. Various vaccination types may lead to incidence of large, medium and small vessel vasculitis including vasculitis of central nerve system. Increasing cases of autoimmune vasculitis following COVID-19 vaccination has been reported.^[6]^

ATM can be a rare adverse event of vaccination against a variety of infections, such as influenza, human papilloma virus and hepatitis A or B.^[7]^ The term “transverse” literally indicates complete involving across the spinal cord. However, APTM as a subtype of ATM has attracted attention due to its radiological characteristics and risk of conversion to multiple sclerosis (MS). APTM is considered having higher risk for relapse than ATM. Bilateral motor, sensory and autonomic dysfunction referable to spinal cord are considered typical manifestation of ATM according to the Transverse Myelitis Consortium Working Group. Cases with ATM associated with SARS-CoV-2 infection were also reported to predominantly present bilateral neurological defects.^[8]^ ATM is recognized as a serious adverse event of COVID-19 vaccination. The trial of ChAdOx1 nCoV-19 vaccine (AZD1222) reported a case of ATM in the experimental group which was assessed “possibly related” to vaccination.^[9]^

A pooled analysis involving various COVID-19 vaccine data from 8 countries indicated that the incidence of post-vaccination ATM is “very rare” in each age and sex subgroup.^[10]^ Interestingly, a study using prediction models on basis of observed incidences also suggested rare events of ATM in subjects receiving COVID-19 vaccines, with essentially lower incidence than that of other demyelinating conditions including Guillain–Barré syndrome, encephalopathy, optic neuritis and Bell’s palsy.^[11]^

Partial TM episode can be an initial sign of MS. Demyelinating lesion in brain and positive OCB in CSF are proposed to be predictive factors for conversion to MS. However, the overall rate of conversion to MS in APTM patients with normal cerebral MRI is low. As an immunological hallmark, OCB is not specific for MS. It can also be seen in other inflammatory conditions and infections of the central nervous system (CNS). Presence of OCB can contribute to the impression of inflammatory diseases although it cannot exclude non-inflammatory ones such as ischemic and hemorrhagic stroke. It was estimated that OCB presented in 38% of APTM with unknown etiology.^[12]^ In this case, the presence of OCB also suggested an inflammatory lesion in the spinal cord. In addition to the radiological characteristics and CSF analysis, the patient’s good responsiveness to corticosteroids treatment also suggested inflammation.

## 4. Conclusion

To the best of our knowledge, this is the first case reporting the concurrence of possible vasculitis in vertebral bodies and APTM following COVID-19 vaccination. Both vasculitis and APTM are rare autoimmune adverse events following COVID-19 vaccination according to current data. Especially for APTM, the incidence should be aware of when encountering asymmetric neurological symptoms after vaccination. Repetitive MRI, comprehensive CSF analysis containing an OCB item and autoimmune antibodies may have particular value for the establishment of diagnosis.

## Author contributions

**Investigation:** Yanyi Chen, Tao Zhan.

**Methodology:** Yanyi Chen, Tao Zhan.

**Project administration:** Tao Zhan.

**Resources:** Yuxin Li, Tao Zhan.

**Visualization:** Yuxin Li.

**Writing – original draft:** Yanyi Chen.

**Writing – review & editing:** Tao Zhan.
